# Editorial: Exploring sulfur compounds’ role in inflammation and therapeutic potential

**DOI:** 10.3389/fimmu.2026.1804564

**Published:** 2026-02-12

**Authors:** Małgorzata Iciek, Tomohiro Sawa, Eugenia Rita Lauriano, Magdalena Kotańska

**Affiliations:** 1 Chair of Medical Biochemistry, Jagiellonian University, Medical College, Krakow, Poland; 2 Department of Microbiology, Graduate School of Medical Sciences, Kumamoto University, Kumamoto, Japan; 3 Department of Chemical, Biological, Pharmaceutical, and Environmental Sciences, University of Messina, Messina, Italy; 4 Laboratory of Pharmacological Screening, Department of Pharmacodynamics, Jagiellonian University Medical College, Krakow, Poland

**Keywords:** hydrogen sulfide, inflammation, reactive sulfur species, sulfur dioxide, supersulfides

Sulfur, long recognized as an essential element for life, has moved from the periphery of environmental science to the center of modern redox biology. Recent research has emphasized the role of reactive sulfur species (RSS), such as hydrogen sulfide (H_2_S), hydropersulfides (RSSH), polysulfides (RSS_n_R) as well as sulfur dioxide (SO_2_) in numerous biological processes (1, 2). The main biological mechanism of RSS action is protein persulfidation—reversible modification of cysteine -SH groups into -SSH residues (3). In turn, SO_2_, which is emerging as a novel gasotransmitter can be responsible for protein sulfenylation (-SOH) (2). Given the participation of RSS in various physiological and pathological pathways, their role in immune homeostasis is being extensively studied (4, 5). This Research Topic of *Frontiers in Immunology* explores the immunomodulatory mechanisms of RSS, bringing together noteworthy research and reviews that elucidate how these species navigate complex signaling pathways to maintain homeostasis. Furthermore, it highlights new avenues for treating chronic and acute inflammatory diseases through regulation of RSS homeostasis.

The review article by Chen et al. provides a comprehensive overview of the latest findings regarding the role of RSS in the immune system, highlighting their context-dependent, bidirectional effects governed by cellular redox states, as well as metabolic and inflammatory conditions. Following a general introduction to RSS biosynthesis, chemical properties, and biological importance, the authors focus on the mechanisms regulating key signaling pathways. The authors point out that RSS regulate immune cells through the persulfidation of NF-κB and Keap1, maintain mitochondrial integrity, and control the metabolic switch between glycolysis and mitochondrial respiration. Furthermore, RSS can inhibit inflammation and autoimmune diseases by suppressing Neutrophil Extracellular Traps (NETosis) through the ROS-MAPK-PAD4 signaling cascade. The authors conclude their manuscript by identifying knowledge gaps and challenges, including the specificity of RSS and the dose-response relationship. Moreover, the therapeutic potential of RSS donors for the treatment of immune-related diseases is discussed.

The next excellent review paper, by Zhang et al., shifts the focus to supersulfides, such as hydropersulfides (RSS_n_H) and polysulfides (RSS_n_R). These molecules, characterized by catenated sulfur chains, are now recognized as potent immunomodulators capable of managing inflammatory responses effectively. They are primarily generated through the activity of cysteinyl-tRNA synthetase (CARS), particularly the mitochondrial CARS2 isoform, and execute their functions through S-persulfidation. The authors highlight how supersulfide donors exert anti-inflammatory effects by suppressing NF-κB signaling, modulating the NLRP3 inflammasome, and blocking JAK/STAT pathways, which leads to the inhibition of interferon responses in macrophages. Furthermore, the authors discuss the development of various supersulfide donors designed to deliver RSS to specific tissues. These donors show promise in treating a range of conditions, including sepsis, rheumatoid arthritis, and metabolic-associated fatty liver disease (MAFLD). Moreover, the review examines the potential role of supersulfides in treating COVID-19 and influenza by inactivating viral proteases and dampening the “cytokine storm”.

The final review paper in this Research Topic, by Hirabayashi et al., is dedicated to the enzymes that regulate intracellular RSS levels. The authors focus not only on the enzymes involved in the production of H_2_S and supersulfides including cystathionine β-synthase (CBS), cystathionine γ-lyase (CSE), 3-mercaptopyruvate sulfurtransferase (3MST), and CARS—but also on those responsible for their catabolism, namely sulfide:quinone oxidoreductase (SQOR), persulfide dioxygenase (ETHE1), and thiosulfate sulfurtransferase (TST). The manuscript provides a comprehensive overview of their roles in maintaining RSS homeostasis and their enzymatic reaction mechanisms. While this review is not exclusively focused on inflammatory processes, it provides valuable information regarding selective inhibitors targeting each of the individual enzymes involved in RSS regulation. As the authors emphasize, these inhibitors could serve as powerful tools for elucidating the biological functions of RSS in inflammation and may hold significant therapeutic potential.

The original paper by Golenkina et al. explores how disulfide stress is linked to leukotriene synthesis. It is well-established that neutrophils are the primary immune cells recruited to the site of invading pathogens. When neutrophils interact with bacteria, they produce leukotriene B4 (LTB_4_), a powerful chemoattractant that, along with the principal bacterial chemoattractant i.e. N-formylmethionine-leucine-phenylalanine (fMLP), promotes the formation of neutrophil clusters around pathogens. Golenkina et al. investigated the regulating role of H_2_S in neutrophil cellular responses using an experimental model of neutrophils interacting with *Salmonella typhimurium*. Their findings demonstrate that under conditions of disulfide stress, which occurs during neutrophil-bacteria interactions, the use of the H_2_S donor supports the synthesis of LTB_4_, with a subsequent increase in the accumulation of neutrophils in bacterial clusters. This discovery can be used to search for innovative therapeutic interventions in bacterial infection states using H_2_S donors.

The next interesting finding in this Research Topic is related to the role of SO_2_ in mast cell activation-related diseases. It is known that mast cell degranulation is a critical step in allergic and inflammatory responses. Endogenous SO_2_ is an essential regulator of mitochondrial activity and is involved in the inflammatory response; however, the mechanism underpinning its participation in mast cell degranulation remained unknown until recently. The study by Song et al. elucidated that SO_2_ inhibits mast cell degranulation via the sulfenylation of galectin-9 (Gal-9) at cysteine 74 under both physiological and IgE- and non-IgE-stimulated pathophysiological conditions. This study identifies Gal-9 as an important regulatory molecule for controlling immune cell activity, and a biological target for therapeutic interventions. The development of drugs that stimulate sulfenylation of Gal-9 might provide a novel treatment approach for mast cell activation-related diseases.

In conclusion, we believe that this Research Topic provides a profound exploration of the role of sulfur compounds in inflammation and offers a critical discussion of their therapeutic potential. In the published papers, the authors have presented the most up-to-date information on the roles of reactive sulfur species (RSS)—primarily H_2_S, persulfides, polysulfides, and SO_2_—in inflammatory diseases. It should be emphasized that the biological effects of RSS are bidirectional and context-dependent. Specifically, they can either promote or suppress immune responses, depending on their concentration and the cellular redox environment. These factors must be carefully considered in future research and in the establishment of dose-response relationships for clinical applications.
